# Hypohydration *per se *affects mood states and executive cognitive processing: results from a face-valid model for studying some consequences of 'voluntary dehydration'

**DOI:** 10.1186/2046-7648-4-S1-A97

**Published:** 2015-09-14

**Authors:** Toby Mündel, Stephen Hill, Stephen Legg

**Affiliations:** 1 School of Sport and Exercise, Massey University, New Zealand; 2 School of Psychology, Massey University, New Zealand; 3 School of Public Health, Massey University, New Zealand

## Introduction

There is limited literature on the effects of a deficit in body water on human cognitive function, with inconsistent and contradictory results. In his critical review of this area Lieberman [1] recommended that typical confounding factors such as the method to induce dehydration (exercise, heat stress, diuretics) and other experimental control (sleep, diet, caffeine) should be considered carefully as little existing research has done so. Few studies have actually assessed what occurs naturally, namely a person simply not drinking sufficiently. The purpose of this study was to measure the cognitive effects of 'not drinking enough' ('voluntary dehydration') when other confounding factors such as sleep, diet and caffeine are controlled.

## Methods

Using a randomized, cross-over design in a stable laboratory environment (20 °C, 600 lx), 24 males (26 ± 6 y) were given at least three familiarisation sessions, followed by two experimental sessions where measures of hydration (body mass and urine specific gravity), mood states (POMS) and aspects of cognition (logical reasoning, working memory, executive processing) were assessed following 24-h of similar diet, sleep and caffeine intake where only fluids consumed differed: usual *ad libitum *(euhydration) vs. complete restriction (hypohydration). Data were assessed using paired samples *t*-test and are reported as mean ± standard deviation.

## Results

Sleep duration (8.5 ± 1.1 h), caloric (9391 ± 3806 kJ), macronutrient (46 ± 11 % CHO, 22 ± 8 % PRO, 31 ± 9 % FAT) and caffeine intake (54 ± 28 mg) were similar (all *p *> 0.05) whilst water intake was lower with hypohydration (285 ± 446 mL) compared to euhydration (1436 ± 1311 mL). This led to a reduction in body mass of 1.2 ± 0.9 kg or 1.4 ± 1.1 % with hypohydration (*p *< 0.001). Similarly, urine specific gravity increased from 1.015 ± 0.010 to 1.024 ± 0.004 (*p *< 0.001) with hypohydration. For mood states, all the derived factors were detrimentally affected with hypohydration (all *p *< 0.01). Performance of working memory and executive processing (both *p *< 0.05) were adversely affected with hypohydration, but logical reasoning was not (*p *> 0.05).

## Discussion

In populations (e.g. illness/disease, ageing) or occupations (e.g. aviation, military) where voluntary dehydration may be common these findings indicate that even mild hypohydration (1.4%), typical of a working day, may have detrimental consequences for mood and some executive cognitive functions.

## Conclusion

Having controlled several confounding factors (exercise, heat stress, diuretics, sleep, diet, caffeine) that previous studies have not, this study indicates that hypohydration *per se *can negatively affect mood states, working memory and executive processing performance but not logical reasoning.
